# Evaluation of *in vitro* Antifungal Activity of *Xylosma prockia* (Turcz.) Turcz. (Salicaceae) Leaves Against *Cryptococcus* spp.

**DOI:** 10.3389/fmicb.2019.03114

**Published:** 2020-02-06

**Authors:** Mariany L. C. Folly, Gabriella F. Ferreira, Maiara R. Salvador, Ana A. Sathler, Guilherme F. da Silva, Joice Castelo Branco Santos, Julliana R. A. dos Santos, Wallace Ribeiro Nunes Neto, João Francisco Silva Rodrigues, Elizabeth Soares Fernandes, Luís Cláudio Nascimento da Silva, Gustavo José Cota de Freitas, Ângelo M. Denadai, Ivanildes V. Rodrigues, Leonardo M. Mendonça, Andrea Souza Monteiro, Daniel Assis Santos, Gabriela M. Cabrera, Gastón Siless, Karen L. Lang

**Affiliations:** ^1^Multicentric Program in Biochemistry and Molecular Biology, Federal University of Juiz de Fora, Governador Valadares, Brazil; ^2^Department of Pharmacy, Federal University of Juiz de Fora, Governador Valadares, Brazil; ^3^Post Graduate Program, CEUMA University, São Luís, Brazil; ^4^Institute of Biological Sciences, Federal University of Minas Gerais, Belo Horizonte, Brazil; ^5^Department of Organic Chemistry, UMYMFOR-CONICET, FCEN, University of Buenos Aires, Buenos Aires, Argentina

**Keywords:** *Cryptococcus* spp., *Xylosma prockia*, antifungal agents, natural products, biodiversity

## Abstract

*Cryptococcus* species are responsible for important systemic mycosis and are estimated to cause millions of new cases annually. The available therapy is limited due to the high toxicity and the increasing rates of yeast resistance to antifungal drugs. Popularly known as “sucará,” *Xylosma prockia* (Turcz.) Turcz. (Salicaceae) is a native plant from Brazil with little information on its pharmacological potential. In this work, we evaluated *in vitro* anticryptococcal effects of the leaf ethanolic extract of *X. prockia* and its fractions against *Cryptococcus gattii* and *Cryptococcus neoformans*. We also evaluated phenotypic alterations caused by ethyl acetate fraction (EAF) (chosen according to its biological results). The liquid chromatography–mass spectrometry (LC-MS) analysis of EAF demonstrated the presence of phenolic metabolites that belong to three structurally related groups as majority compounds: caffeoylquinic acid, coumaroyl-glucoside, and caffeoyl-glucoside/deoxyhexosyl-caffeoyl glucoside derivatives. The minimum inhibitory concentration (MIC) values against *C. gattii* and *C. neoformans* ranged from 8 to 64 mg/L and from 0.5 to 8 mg/L, for ethanolic extract and EAF, respectively. The EAF triggered an oxidative burst and promoted lipid peroxidation. EAF also induced a reduction of ergosterol content in the pathogen cell membrane. These effects were not associated with alterations in the cell surface charge or in the thermodynamic fingerprint of the molecular interaction between EAF and the yeasts evaluated. Cytotoxic experiments with peripheral blood mononuclear cells (PBMCs) demonstrated that EAF was more selective for yeasts than was PBMCs. The results may provide evidence that *X. prockia* leaf extract might indeed be a potential source of antifungal agents.

## Introduction

In recent years, the incidence of opportunistic mycosis has increased significantly, becoming an important public health problem (Herkert et al., 2017). Cryptococcosis is an important systemic mycosis caused by fungi of the genus *Cryptococcus*, mainly by *Cryptococcus neoformans* and *Cryptococcus gattii*, responsible for approximately one million new cases and 600,000 deaths annually (Maziarz and Perfect, 2016). Cryptococcal meningitis is the most severe form of the disease and remains a major problem in resource-limited countries, where HIV prevalence is high and access to healthcare is limited. Worldwide, nearly 220,000 new cases of cryptococcal meningitis occur each year, resulting in 181,000 deaths (Rajasingham et al., 2017). In this context, immunocompromised patients, such as the elderly and individuals with chronic pathologies, are at high risk of becoming ill with cryptococcosis (May et al., 2016).

The current therapeutic options for treating these mycoses are restricted due to the increased resistance of yeasts to the available drugs, as well as the high toxicity of some, such as amphotericin B (AMB) (Bongomin et al., 2018; Wiederhold, 2018).

Several strategies and techniques are currently available to assist in drug discovery and development, and natural products represent one of the most successful alternatives (Davison and Brimble, 2019). Different studies have investigated the activity and efficacy of plant extracts/fractions (Thammasit et al., 2018) and their secondary metabolites (Alves et al., 2017; Li et al., 2017) against *Cryptococcus* spp. These have indicated that plant-derived preparations and compounds act in such pathogens by targeting their survival and virulence, increasing host defense or enhancing the activity of known antifungal drugs. However, the antifungal potential of many plant species have not yet been evaluated.

*Xylosma prockia* (Turcz.) Turcz. (Salicaceae) is a native species from Brazil (found mainly in the northeast, southeast, and south), popularly known as “sucará” (Longhi et al., 2006). The few existing studies on this plant are restricted to its morphology and taxonomy and have not addressed its chemical constitution and pharmacological activities. Flavonoids, triterpenes, coumarins, and phenolic glycosides have been described in other species of the genus *Xylosma* (Parveen and Ghalib, 2012; Devi et al., 2013). In addition, reports have demonstrated antifungal, antibacterial, antispasmodic, narcotic, and sedative properties for extracts and compounds isolated from some *Xylosma* spp. (Mosaddik et al., 2004; Castro et al., 2008; Devi et al., 2013).

In this work, the antifungal activity of *X. prockia* leaf ethanolic extract (EE) and fractions was investigated for the first time against *C. neoformans* and *C. gattii*. The possible effects of *X. prockia* on the pathogen phenotype were also investigated.

## Materials and Methods

### Plant Material and Extraction

The leaves of *X. prockia* (Turcz.) Turcz. (Salicaceae) were collected in Governador Valadares (18°51′04″S, 41°56′58″W), Minas Gerais, Brazil, in December 2015. The sample was identified by the botanist Dr. Ronaldo Marquete and deposited in the RB Herbarium of Rio de Janeiro Botanical Garden, Rio de Janeiro, Brazil (voucher specimen number RB 773293), in August 2018. The research was authorized by the National System for the Management of Genetic Heritage and Associated Traditional Knowledge (SISGEN; no. A66F830).

The leaves were dried at 40°C in an air-circulating oven, and the powdered leaves (340 g) were extracted by maceration in 99.8% ethanol for 5 days (plant: solvent, 1:10, w/v; at room temperature). The organic solvent was evaporated under reduced pressure in a rotary evaporator (temperature below 45°C) to obtain the EE (68.2 g, 20.1%). Subsequently, the EE has undergone a sequential liquid–liquid extraction with organic solvents of increased polarity in the following order: *n*-hexane (HF, 14 g, 20.5%), dichloromethane (DF, 3 g, 4.4%), ethyl acetate (EAF, 8 g, 11.7%), and *n*-butanol (BF, 6 g, 8.8%).

### Liquid Chromatography–Mass Spectrometry (LC-MS) Analysis

The LC-MS analyses were performed in an RRLC Agilent 1200 using a Luna C_18_ column (3 μm, 2.0 × 100 mm; Phenomenex, Torrance, CA, United States). The mobile phase consisted of 85% water containing 0.1% formic acid (A) and 15% acetonitrile (B). The flow rate was 0.3 ml/min, and the column temperature was set to 30°C. Detection was performed with a diode array detector (DAD) from 190 to 950 nm coupled to a mass spectrometer.

Mass spectrometric analyses were performed in a Bruker micrOTOF-Q II mass spectrometer (Bruker Daltonics, Billerica, MA, United States), equipped with an electrospray source using the negative mode. The instrument was operated at a capillary voltage of 4.5 kV with an end-plate offset of 500 V, dry temperature of 200°C using N_2_ as dry gas at 6.0 L/min, and a nebulizer pressure of 3.0 bars. Multipoint mass calibration was carried out using a sodium formate solution from m/z 50 to 1,200 in the negative ion mode. Data acquisition and processing were carried out using the software Bruker Compass Data Analysis version 4.3 supplied with the instrument.

### *Cryptococcus* Strains and Study Design

We initially assessed the antifungal activity of EE and its fractions against two strains of *C. gattii* and two strains of *C. neoformans*. The ethyl acetate fraction (EAF) presented better antimicrobial activity and yield; therefore, it was chosen for this study.

For the “antifungal drug susceptibility testing” assays, we tested two reference strains of *C. gattii* [American Type Culture Collection (ATCC) 24065 and ATCC 32608] and four reference strains of *C. neoformans* (ATCC 24067, ATCC 28957, ATCC 62066, and ATCC H99), which were obtained from the Culture Collection of the University of Georgia (Atlanta, GA, United States). Seven clinical isolates of *C. gattii*, five clinical isolates of *C. neoformans*, and one environmental isolate of each species, all from the Culture Collection of the Mycology Laboratory/ICB-UFMG, were also used in this study (Magalhães et al., 2013). Isolates were maintained on Sabouraud dextrose broth (SDB) at −80°C. Prior to each test, the strains were subcultured on Sabouraud dextrose agar (SDA) for 48 h at 35°C.

The ATCC 32608 and L27/01 of *C. gattii* and ATCC 2895 and ATCC H99 of *C. neoformans* strains were randomly chosen for further experiments, except for ITC, in which we used ATCC 32068 and ATCC H99 strains.

### Antifungal Drug Susceptibility Testing

The minimum inhibitory concentrations (MICs) for EE and its fractions were determined by the antifungal microdilution susceptibility standard test, proposed by the CLSI M27-A3 method (Institute Clinical and Laboratory Standards [CLSI], 2008). The inoculum was prepared in sterile saline, and the transmittance of suspensions was adjusted to 75–77% (530 nm), followed by further dilution in RPMI 1640 buffered with MOPS (Sigma-Aldrich^®^) medium to achieve 1.0–5.0 × 10^3^ colony-forming unit (CFU)/ml. The final concentrations of EE and fractions ranged from 0.25 to 128 mg/L, from 0.125 to 64 mg/L for fluconazole (FLC) (Sigma-Aldrich^®^), and from 0.03 to 16 mg/L for AMB (Sigma-Aldrich^®^). The plates were incubated at 35°C for 72 h. The MIC was determined visually as 100% growth inhibition when compared to the control, except for FLC, in which the MIC was determined visually as 50% growth inhibition, when compared to the control. The results were confirmed through the assessment of fungal metabolic activity by adding 3-(4,5-dimethylthiazol-2-yl)-2,5-diphenyl-2*H*-tetrazolium bromide (MTT) (Sigma-Aldrich^®^) (5.0 mg/ml). For this, the plates were incubated at 35°C for 3 h, and DMSO was added before spectrophotometric reading at 570 nm.

The minimal fungicidal concentration (MFC) was defined as the concentration of the antifungal agent in which the number of CFUs was zero. For determining the MFC, at the end of the MIC experiments, the samples (10 μl) were removed from all wells of the standard MIC plates and placed on Petri plates containing SDA (Difco^®^). The plates were incubated for 72 h at 35°C before the colonies were counted. All tests were performed in duplicate and repeated three times.

### Time–Kill Curves

An assay was performed to evaluate time–kill kinetics of EAF against *C. gattii* (ATCC 32068 and L27/01) and *C. neoformans* (ATCC 28957 and ATCC H99) strains as previously described by Ahmad et al. (2011), with modifications. A 100-μl inoculum of yeasts (1.0–5.0 × 10^3^ CFU/ml) was placed on microtiter plates at different concentrations of EAF (MIC, 2 × MIC, and 4 × MIC) and incubated at 35°C for 72 h. A control growth was performed at 0, 3, 6, 12, 24, 36, 48, and 72 h. Aliquots of 100 μl were removed from each test and plated on SDA (Difco^®^). For control growth, the aliquots were diluted in saline solution prior to plating. Colony counts were determined after incubation at 35°C for 72 h. The results were expressed as CFUs per milliliter.

### *In vitro* Interaction of FLC and AMB With EAF

The possible interactions between EAF (0.25–128 mg/L) and the commercially used antifungals FLC (0.5–32 mg/L) and AMB (0.03 to 1 mg/L) were investigated *in vitro* by using a checkerboard microdilution assay, as previously described by Santos et al. (2012). The plates were incubated at 35°C for 72 h, and the cellular metabolic activity was determined by the MTT salt.

The interactions were determined by the fractional inhibitory concentration index (FICI) (Odds, 2003). FICI was calculated as (MIC FLC or AMB in combination with EAF/MIC FLC or AMB alone) + (MIC EAF in combination with FLC or AMB/MIC EAF alone). Interactions were classified as synergism if FICI ≤ 0.5, indifference if 0.5 > FICI ≤ 4.0, and antagonism if FICI > 4.0. This assay was tested in duplicate and repeated twice.

### Measurement of Reactive Oxygen Species (ROS) Production

The endogenous production of ROS and peroxynitrite by fungal cells was measured by fluorometry (Synergy 2 SL Luminescence Microplate Reader; BioTek^®^) with specific probes (Ferreira et al., 2013). The cells (1.0 × 10^3^ to 5.0 × 10^3^ cells per milliliter) were incubated with EAF (MIC and 2 × MIC) or AMB (MIC) in RPMI 1640 without phenol red (Sigma-Aldrich^®^) containing 10 mM 2’,7’-dichlorofluorescein diacetate (for ROS quantification; Invitrogen^®^) or 20 mM dihydrorhodamine 123 (for peroxynitrite quantification; Sigma-Aldrich^®^). The fluorescence was measured 24 h later at 500 nm. At the end of the experiments, 10 μl of each sample was placed on SDA-containing plates, and the numbers of CFU were counted. The results are expressed as arbitrary units of fluorescence/CFU.

### Measurement of Mitochondrial Membrane Potential

The cells (1 × 10^6^ cells per milliliter, in 500 μl) were treated with EAF (MIC) for 24 h, at 37°C. After being washed, the cell pellets were resuspended in phosphate-buffered saline (PBS) (500 μl) and labeled with rhodamine 123 (Rho 123) (10 μg/ml in the dark for 10 min) (Ronot et al., 1986; Olsson et al., 1987; Alves et al., 2017). The cells were washed three times, resuspended in PBS, and analyzed by flow cytometry (BD Accuri^TM^, United States; FL3 channel for AO and FL1 for Rho 123). A total of 10,000 events were analyzed for each sample. Changes in the fluorescent intensity of Rho 123 were quantified using the variation index (VI) obtained by the equation (MT − MC)/MC, in which MC is the mean of fluorescent intensity of control and MT is the mean of treated cells. The negative values of VI correspond to mitochondrial membrane depolarization.

### Lipid Peroxidation

Thiobarbituric acid-reactive substances (TBARSs) were measured as an index of lipid peroxidation products, as previously described by Soares et al. (2011). *C. neoformans* and *C. gattii* were inoculated into 50 ml of SDB overnight (Difco^®^) containing EAF (MIC and 2 × MIC) or hydrogen peroxide (HP) as a positive control. The cultures were incubated for 24 h. After incubation, the tubes were centrifuged (Jouan, model BR4i) at 2,700 rpm for 5 min at 4°C, and the supernatant was discarded. The precipitates (cells) were washed with sterile distilled water, and their net wet weight was determined. The TBARS values were calculated using the extinction coefficient of 156 L/mol⋅cm. The results were divided by the net weight and expressed as nanomolars per gram.

### Ergosterol Quantification

Total intracellular sterols were extracted as previously described, with modifications (Arthington-Skaggs et al., 1999; Alves et al., 2017). *C. neoformans* and *C. gattii* were inoculated into 50 ml of SDB overnight (Difco^®^), containing MIC or 2 × MIC of EAF or FLC (MIC) as a positive control. The cultures were incubated for 24 h. After incubation, the tubes were centrifuged (Jouan, model BR4i) at 2,700 rpm for 5 min at 4°C, and then the supernatant was removed. The cells were washed with sterile distilled water, and the net wet weight pellet was determined. Three milliliters of a 25% potassium hydroxide alcohol solution (25 g of KOH in 65% ethanol) was added to each pellet and mixed for 1 min. Cell suspensions were transferred to sterile tubes and incubated at 85°C in a water bath for 1 h. After incubation, the tubes were allowed to cool down to room temperature. The sterols were extracted by adding a mixture of 1 ml of sterile distilled water and 3 mL of *n*-heptane followed by vigorous vortexing for 3 min. The supernatant was removed, and the reading was performed in a spectrophotometer at 282 and 230 nm. A calibration curve with standard ergosterol (Sigma-Aldrich^®^) was used to calculate the quantity of ergosterol. In all cases, the absorbance of ergosterol was the result of the subtraction of the absorbance obtained at 282 and 230 nm (Breivik and Owades, 1957). The results were divided by net weight and expressed as mg/L⋅g^–1^.

### Sorbitol Test

The sorbitol protection assay was carried out by the modified CLSI M27-A3 protocol as described above (Lee and Kim, 2016). Briefly, one plate was prepared containing EAF ranging from 0.25 to 128 mol/L and another plate containing EAF ranging from 0.25 to 128 mol/L plus 0.8 M of sorbitol as osmotic protectant. The plates were incubated at 35°C for 72 h. The reading was performed visually. All tests were conducted in duplicates for each strain.

### Carboxyfluorescein Succinimidyl Ester (CFSE) Assay

*Cryptococcus gattii* and *C. neoformans* were grown in RPMI supplemented with 0.5 × MIC of EAF or with no drugs for 24 h at 37°C, washed in PBS, and stained with 25 μg/ml CFSE (Sigma-Aldrich^®^) for 30 min at 30°C. The yeasts were washed in PBS containing 2% bovine serum albumin (BSA) to remove excessive CFSE. Stained yeast cells were passed through a 25-G 7/8-in. needle to dissociate clumped cells. Flow acquisition was performed with a FACSCalibur flow cytometer (Becton-Dickinson^®^), using the CellQuest software (Becton-Dickinson^®^). A total of 10,000 events were analyzed for each sample. The results are expressed as arbitrary units of fluorescence.

### Cell Diameter, Capsule Size, and Zeta Potential (ZP) Measurements

Yeasts cells cultured with 0.5 × MIC of EAF were visualized with an optical microscope (Axioplan, Carl Zeiss^®^) following suspension in India ink. The capsule and the diameter of at least 100 cells were measured using the ImageJ 1.40g software^1^ (National Institutes of Health, NIH, Bethesda, MD, United States). The surface-to-volume ratio (*S*/*V*) was calculated using the formula 3/*r*, in which *r* is the radius (Ferreira et al., 2015).

Zeta Potential experiments were performed using a Malvern Zetasizer Nano ZS equipment (Ferreira et al., 2016). The ZP was determined by a laser Doppler microelectrophoresis technique, at a scattering angle of 173°, using a disposable cell folded capillary (DPS1060). Zeta Plus software was used for ZP (Brookhaven Instruments Corp., Holtsville, NY, United States). ZP values were calculated as the average of 10 independent measurements, each obtained as the mean of 30 counts.

### Isothermal Titration Calorimetry (ITC)

Isothermal Titration Calorimetry experiments were carried out with one repetition using a VP-ITC microcalorimeter (MicroCal, LLC, Northampton, MA, United States) at 25°C, after previous electrical and chemical calibration (Monteiro et al., 2011). All the solutions employed in the experiment were previously degasified under vacuum (140 mbar) during 8 min. Each titration experiment consisted of 51 successive injections of 5 μl of EAF at 1,000 mg/L into a chamber containing 1.5 ml of *C. gattii* and *C. neoformans* suspension at 1 × 10^6^ CFU/ml. The first 1-ml injection was discarded to eliminate diffusion effects of the syringe material to the calorimetric chamber. The injection time was 2 s, and the interval between the injections was 240 s.

### Human PBMC Viability

The MTT colorimetric assay, as proposed by Mosmann (1983), was used to determine the PBMC viability when grown with EAF. PBMCs were collected from eight healthy human volunteers (non-smoking donors who had not received any medication for the last 15 days prior to sampling, aged 18–35 years old) who provided written formal consent. This study was approved by the Research Ethics Committee of the Federal University of Juiz de Fora (protocol number: 70972117.0.0000.5147). The cells were obtained by the standard method of density gradient centrifugation over Histopaque R-1119 according to the manufacturer’s instructions. PBMCs were suspended in a supplemented RPMI 1640 culture medium (Life Technologies^®^) containing 10% fetal bovine serum (Life Technologies^®^), streptomycin (100 μg/ml; Sigma-Aldrich^®^), and penicillin (100 U/ml; Sigma-Aldrich^®^).

In a 96-well plate, 100 μl of PBMCs at a density of 1 × 10^6^ cells per milliliter suspended in RPMI 1640 medium was added. After 24 h of incubation at 37°C in CO_2_, the cells were incubated with 100 μl of different concentrations of EAF (4–512 μg/ml; in RPMI 1640 medium), in CO_2_ (5%), at 37°C, for 24 h. Then, 10 μl of MTT solution (5 mg/ml; Sigma-Aldrich^®^) was added to all wells of the plate, which was incubated for 4 h. After the incubation period, MTT medium was carefully removed from all wells, and 100 μl of DMSO (Nuclear^®^) was added to solubilize formazan. The plates were gently shaken at room temperature for 5–10 min and read at 540 nm in an ELISA reader (Biochrom Asys Expert Plus^®^). This assay was tested in duplicate and repeated three times.

### Statistical Analysis

The results were expressed as mean ± standard error (SE), and *P*-values ≤ 0.05 were considered statistically significant. All statistical analyses were performed using GraphPad Prism version 6.00 for Windows (GraphPad Software, San Diego, CA, United States). Comparisons between two groups were conducted by Student’s *t*-test (parametric data) or the Mann–Whitney test (non-parametric data). Multiple comparisons were performed by one-way analysis of variance (ANOVA) followed by Dunn’s test.

## Results

### Characterization of Antifungal Effects of Leaf EE and EAF of *X. prockia*

**FIGURE 1:**
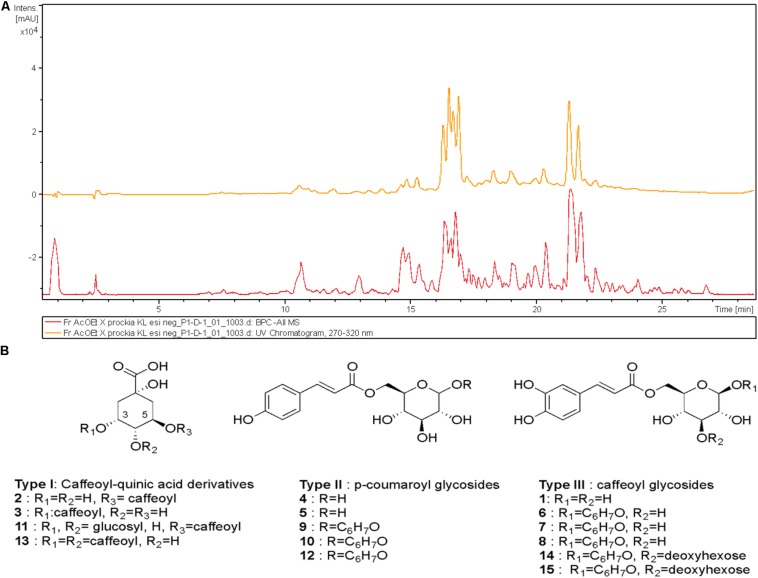
High-performance liquid chromatography–ultraviolet (HPLC-UV) (270–320 nm, upper trace) and mass spectrometry (MS) (base peak chromatogram, lower trace) profile of ethyl acetate fraction (EAF) of *Xylosma prockia* leaves in the negative mode (−) **(A)**. Detected compounds in EAF of *X. prockia* in electrospray ionization (ESI) (−) **(B)**.

**FIGURE 2:**
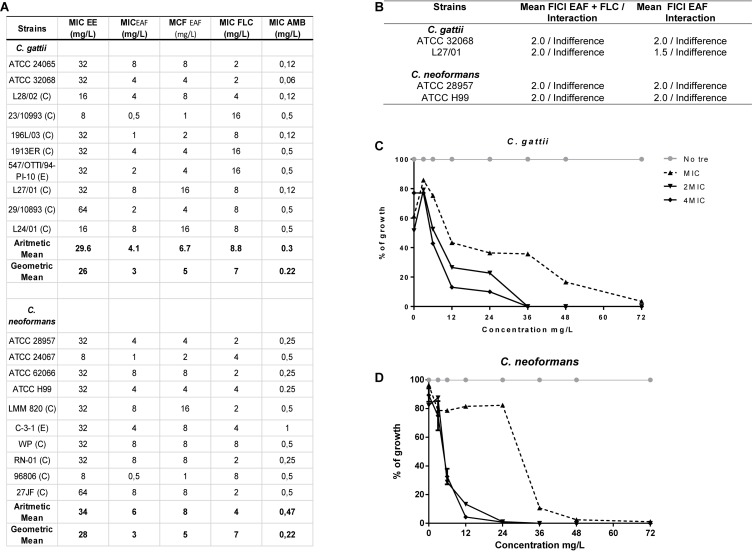
Screening of the antimicrobial effects of *Xylosma prockia* leaves. Table listing the MIC of ethanolic extract (EE), ethyl acetate fraction (EAF), fluconazole (FLC), and amphotericin B (AMB) plus the MFC of EAF against *Cryptococcus gattii* and *Cryptococcus neoformans* **(A)**. Table showing mean of fractional inhibitory concentration index (FICI) and interaction between antifungal drugs and EAF against two strains of *C. gattii* and *C. neoformans* **(B)**. Time–kill curves generated against two strains of *C. gattii* **(C)** and *C. neoformans* **(D)** at different concentrations of EAF. The results of the time–kill curve are expressed as the percentage of growth compared with growth of the control. Table showing mean of fractional inhibitory concentration index (FICI) and interaction between antifungal drugs and EAF against two strains of *C. gattii* and *C. neoformans* **(D)**. ATCC, American Type Culture; E, Environmental; C, Clinical; MIC, Minimal inhibitory concentration; MFC, Minimal fungicidal concentration.

*Xylosma prockia* EE presented MIC values from 8 to 64 mg/L against *C. gattii* and *C. neoformans* (Figure 1A). Among the tested fractions, EAF exhibited the best results (higher yield and lower values of MIC) (Supplementary Table S1) and was chosen for the experiments. The EAF was effective against both *C. gattii* and *C. neoformans*, with MIC values of 0.5–8 mg/ml and MFC from 1 to 8 mg/ml (Figure 1A). Similar results were obtained for FLC (MIC of 2–16 mg/L) (Figure 2A).

The time-dependent effects of EAF on *C. gattii* and *C. neoformans* viability were also investigated in a time–kill assay (Figures 2C,D). EAF MIC reduced 97.5% of *C. gattii* and 99% of *C. neoformans* growth when incubated for at 72 and 36 h, respectively. *C. gattii* growth was reduced by 100% following 36 h of exposure to EAF at 2 × MIC and 4 × MIC. A similar effect was observed for *C. neoformans* 24 h post incubation with EAF.

Ethyl acetate fraction was also tested in combination with FLC and AMB, drugs usually chosen to treat cryptococcosis (Mourad and Perfect, 2018). No interactions were observed between EAF and these antifungal drugs when assessed against *C. gattii* (ATCC 32068 and L27/01) and *C. neoformans* strains (ATCC 28957 and ATCC H99) (Figure 2B).

The high-performance liquid chromatography–mass spectrometry (HPLC-MS) analysis of the EAF of *X. prockia* obtained using electrospray ionization (ESI) in negative ionization mode allowed visualization of 15 major peaks (Figure 1), which were assembled in three distinct groups of compounds (I, caffeoylquinic acid derivatives; II, coumaroyl-glucoside derivatives; III, caffeoyl-glucoside/deoxyhexosyl-caffeoyl glucoside derivatives). Based on the fragmentation pattern (Supplementary Figure S2), type I compounds presented peaks corresponding to characteristic fragments of quinic acid at m/z 191 and caffeic acid at m/z 179 and m/z 161. Additionally, the observed product ions in each MS/MS spectrum allowed the isomeric differentiation of some compounds (Table 1). Type II and compounds **6–8** were identified as 6-*p*-coumaroyl-glucose or 6-caffeoyl-glucose derivatives, respectively, based on key fragmentation patterns (m/z 205 ions for coumaroyl and m/z 221 for caffeate derivatives). The compounds having the highest relative abundance in the chromatogram showed [M–H]^–^ at m/z 419.1342, corresponding to a molecular formula of C_21_H_24_O_9_, being consistent with the presence of coumaroate, glucose, and an additional residue, C_6_H_7_O, in the metabolites. Although similar compounds with a C_6_ residue attached to the sugar have been reported (Feistel et al., 2017), the preliminary spectroscopic data would indicate that they have different structures and that further isolation would be mandatory to assess the complete characterization of these isomeric compounds (**6**, **7**, and **8**; and **9**, **10**, and **12**).

**TABLE 1:** Hydroxycinnamic acid derivatives identified by high-performance liquid chromatography (HPLC)–electrospray ionization (ESI)–mass spectrometry (MS) in the ethyl acetate fraction (EAF) of *Xylosma prockia*.

**Number**	**Group**	**Tr (min)**	**Proposed compound**	**Molecular formula**	**[M–H]^–^ (m/z)**	**Calculated [M–H]^–^ (m/z)**	**Error (ppm)**	**Precursor ion**	**Product ions (relative intensity)**	**References**
1	III	10.1	caffeoyl glucose	C_15_H_18_O_9_	341.0876	341.0878	0.5	341	135 (100), 179 (60), 161 (55)	
2	I	10.7	5-caffeoyl-quinic acid (5-CQA)	C_16_H_18_O_9_	353.0894	353.0878	−4.6	353	191 (100), 179 (70), 135 (20)	Ouyang et al., 2018
3	I	10.9	3-caffeoyl-quinic acid (3-CQA)	C_16_H_18_O_9_	353.0862	353.0878	4.7	353	191 (100)	Ouyang et al., 2018
4	II	11.0	coumaroyl-glucose	C_15_H_18_O_8_	325.0920	325.0929	2.9	325	119 (100), 145 (50), 163 (30)	
5	II	12.1	coumaroyl-glucose	C_15_H_18_O_8_	325.0941	325.0929	−3.9	325	119 (100), 145 (50), 163 (30)	
6	III	14.7	caffeoyl-glucoside	C_21_H_24_O_10_	435.1302	435.1297	−1.3	435	281 (60), 179 (80), 161 (100)	
7	III	15.0	caffeoyl-glucoside	C_21_H_24_O_10_	435.1307	435.1297	−2.3	435	281 (60), 179 (80), 161 (100)	
8	III	15.4	caffeoyl-glucoside	C_21_H_24_O_10_	435.1304	435.1297	−1.7	435	281 (60), 179 (80), 161 (100)	
9	II	16.4	coumaroyl-glucoside	C_21_H_24_O_9_	419.1342	419.1348	1.3	419	265 (100), 205 (60), 163 (55), 145 (50), 235 (25)	
10	II	16.6	coumaroyl-glucoside	C_21_H_24_O_9_	419.1350	419.1348	−0.7	419	265 (100), 205 (60), 163 (55), 145 (50), 235 (25)	
11	I	16.8	5-caffeoyl-glucosyl-quinic-acid	C_22_H_28_O_14_	515.1412	515.1406	0.3	515	353 (85), 191 (100), 179 (50), 135 (15)	Jaiswal et al., 2014; Ouyang et al., 2018
12	II	17.3	coumaroyl-glucoside	C_21_H_24_O_9_	419.1364	419.1348	−4.0	419	265 (100), 205 (60), 163 (55), 145 (50), 235 (25)	
13	I	18.3	3,5-dicaffeoylquinic acid	C_25_H_24_O_12_	515.1190	515.1195	0.9	515	353 (100), 335 (1), 191 (30), 179 (56), 173 (72)	Jaiswal et al., 2014
14	III	21.3	caffeoyl deoxyhexosyl glucoside	C_27_H_34_O_14_	581.1867	581.1876	1.3	581	161 (100), 435 (54), 487 (43), 179 (34), 203 (30), 427 (17), 235 (10)	
15	III	21.7	caffeoyl deoxyhexosyl glucoside	C_27_H_34_O_14_	581.1877	581.1876	−0.3	581	161 (100), 435 (54), 487 (43), 179 (34), 203 (30), 427 (17), 235 (10)	

The metabolites of group III, with [M–H]^–^ ion at m/z 581, were identified as 6-caffeoyl-3-deoxyhexosyl glucoside derivatives, based on the product ions observed in their MS/MS spectra, mainly m/z 487 (loss of C_6_H_7_O moiety), 435 (loss of deoxyhexose), and m/z 427 and 346, with a deoxyhexose attached at C-4 of the glucose (Supplementary Figure S1).

### EAF Induces Oxidative Burst and Lipid Peroxidation

**FIGURE 3:**
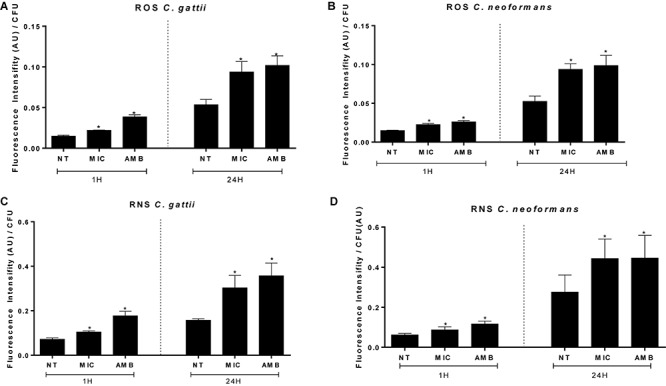
Amounts of reactive oxygen **(A**,**B)** and nitrosative **(C**,**D)** species peroxynitrite induced by EAF and AMB (positive control) against *Cryptococcus gattii* **(A–C)** and *Cryptococcus neoformans* **(B–D)** within 1 and 24 h. The results are expressed in arbitrary units of fluorescence (AU). Data are represented as the mean ± SEM of two independent experiments in triplicate assays. An asterisk represents statistical differences between the treatments and the control (*P* < 0.05). EAF, ethyl acetate fraction; NT, no treatment; AMB, amphotericin B; MIC, minimal inhibitory concentration; ROS, reactive oxygen species; RNS, reactive nitrogen species.

Ethyl acetate fraction resulted in a significant increase of ROS (*C. gattii* 1 h, no treatment: 0.014 ± 0.0003 AU/CFU (arbitrary units of fluorescence/colony forming unit), EAF: 0.021 ± 0.0001 AU/CFU, AMB: 0.038 ± 0.0007 AU/CFU; *C. gattii* 24 h, no treatment: 0.053 ± 0.006 AU/CFU, EAF: 0.093 ± 0.003 AU/CFU, AMB: 0.101 ± 0.003 AU/CFU; *C. neoformans* 1 h, no treatment: 0.014 ± 0.0001 AU/CFU, EAF: 0.022 ± 0.0004 AU/CFU, AMB: 0.026 ± 0.0004 AU/CFU; *C. neoformans* 24 h, no treatment: 0.052 ± 0.001 AU/CFU, EAF: 0.094 ± 0.002 AU/CFU, AMB: 0.098 ± 0.004 AU/CFU; *P* < 0.05) (Figures 3A,B) and peroxynitrite (*C. gattii* 1 h, no treatment: 0.071 ± 0.001 AU/CFU, EAF: 0.010 ± 0.001 AU/CFU, AMB: 0.177 ± 0.005 AU/CFU; *C. gattii* 24 h, no treatment: 0.156 ± 0.002 AU/CFU, EAF: 0.330 ± 0.015 AU/CFU, AMB: 0.357 ± 0.016 AU/CFU; *C. neoformans* 1 h, no treatment: 0.061 ± 0.002 AU/CFU, EAF: 0.086 ± 0.004 AU/CFU, AMB: 0.116 ± 0.004 AU/CFU; *C. neoformans* 24 h, no treatment: 0.275 ± 0.024 AU/CFU, EAF: 0.443 ± 0.027 AU/CFU, AMB: 0.445 ± 0.032 AU/CFU; *P* < 0.05) levels (Figures 3C,D) in *C. gattii* and *C. neoformans* 1 and 24 h following incubation.

**FIGURE 4:**
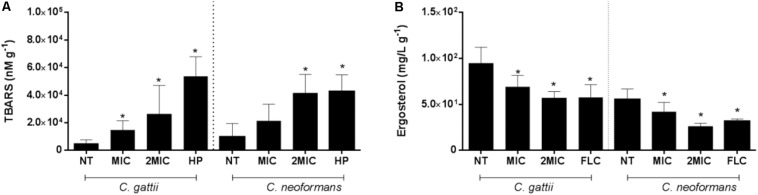
Reduction of lipid peroxidation and ergosterol content are consequences of the treatment with EAF of *Xylosma prockia*. Amount of TBARS in *Cryptococcus gattii* and *Cryptococcus neoformans* cells after 24 h of EAF or HP (positive controls) treatment. The results are expressed in nanomolars per gram **(A)**. Ergosterol levels of cells from *C. gattii* and *C. neoformans* after 24 h of EAF or FLC (positive control) treatments. Results are expressed in micrograms per milliliter per gram **(B)**. Data of these two experiments are represented as the mean ± SEM of two independent experiments in triplicate assays. An asterisk represents statistical differences between the treatments and the control (*P* < 0.05). EAF, ethyl acetate fraction; NT, no treatment; FLC, fluconazole; HP, hydrogen peroxide; MIC, minimal inhibitory concentration; TBARS, thiobarbituric acid-reactive substances.

A similar stimulated effect was observed for EAF on the TBARS levels of *C. gattii* (no treatment: 5,008 ± 1,119 nM⋅g^–1^, MIC EAF: 14,933 ± 2,493 nM⋅g^–1^, 2 × MIC EAF: 26,272 ± 7,396 nM⋅g^–1^, HP: 53,856 ± 6,308 nM⋅g^–1^; *P* < 0.05) (Figure 4A) and *C. neoformans* (no treatment: 9,785 ± 3,667 nM⋅g^–1^, MIC EAF: 21,503 ± 4,504 nM⋅g^–1^, 2 × MIC EAF: 41,563 ± 5,096 nM⋅g^–1^, HP: 42,674 ± 4,563 nM⋅g^–1^; *P* < 0.05) (Figure 4B), in comparison with vehicle-treated cells.

### EAF Does Not Affect Mitochondrial Membrane Potential

Next, we attempted to analyze the effects of EAF in ΔΨm using an assay based on the uptake and retention of Rho 123. The results of cells incubated for 24 h demonstrated no significant differences between EAF-treated cells and cells without treatment (*P* > 0.05) (Supplementary Figure S2).

### EAF Impairs Cell Membrane

Ethyl acetate fraction promotes significant reduction of ergosterol content in *Cryptococcus* cells when treated with 1 × MIC and 2 × MIC of EAF (*C. gattii*, no treatment: 94.78 ± 6.58 mg/L⋅g^–1^, MIC EAF: 69.30 ± 4.371 mg/L⋅g^–1^, 2 × MIC EAF: 56.95 ± 2.85 nM⋅g^–1^, FLC: 53.85 ± 6.30 nM⋅g^–1^; *P* < 0.05) (*C. neoformans*, no treated: 55.39 ± 4.01 mg/L⋅g^–1^, MIC EAF: 42.08 ± 3.97 mg/L⋅g^–1^, 2 × MIC EAF: 26.02 ± 1.38 nM⋅g^–1^, FLC: 31.65 ± 1.04 nM⋅g^–1^; *P* < 0.05) in both species (Figure 4B).

**FIGURE 5:**
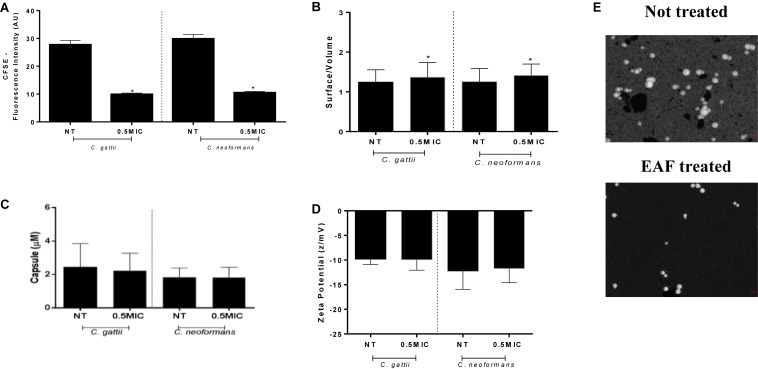
Ethyl acetate fraction stress impairs the cell membrane and increases the surface/volume ratio with no changes on the cell net charge. Cells exposed to 0.5 × MIC of EAF for 24 h were less labeled than the non-treated cells for both species. Data are represented in arbitrary units of fluorescence (AU) **(A)**. Morphometric data are obtained by India ink and are represented by a ratio of surface/volume for cell size **(B)** and micromolars for capsule **(C)**. Zeta potential data are expressed in z/mV **(D)**. Stained cells **(E)**. Data of experiments are represented as the mean ± SEM of two independent experiments in triplicate assays. An asterisk represents statistical differences between the treatments and the control (*p* < 0.05). EAF, Ethyl acetate fraction; NT, No treatment; MIC, Minimal inhibitory concentration; CFSE, Carboxyfluorescein succinimidyl ester.

Interestingly, cells treated with subinhibitory concentrations of EAF had membrane damages, with less CFSE death (*C. gattii*, no treatment: 27.75 ± 1.51 AU, EAF: 9.92 ± 0.44 AU; *P* < 0.05) (*C. neoformans*, no treatment: 29.90 ± 1.52 AU, EAF: 10.50 ± 0.50 AU; *P* < 0.05) (Figure 5A). These results indicate that the EAF causes cell membrane impairment, with a reduction in the amount of ergosterol.

It is important to note that the results of the assays with sorbitol showed no alterations of MIC when *Cryptococcus* cells were exposed to the osmotic protector sorbitol (Lee and Kim, 2016), indicating that damaging effects of the EAF cannot be recovered in the presence of osmoprotectants such as sorbitol.

### Morphological Alterations

Following the results obtained, we researched the ability of *C. gattii* and *C. neoformans* of adapting to the stress caused by EAF through changing morphometric parameters. Morphometric analysis showed that cells exposed to subinhibitory concentrations of EAF have a significantly reduced surface/volume (S/V) ratio for *C. gattii* (no treatment: 1.24 ± 0.02 S/V, EAF: 1.36 ± 0.02 S/V; *P* < 0.05) and *C. neoformans* (no treatment: 1.25 ± 0.02 S/V, EAF: 1.41 ± 0.02 S/V; *P* < 0.05) (Figures 5B,E) strains. No alterations for capsule size measurements (*P* > 0.05) were observed (Figure 5C).

No changes were observed in the cellular superficial charges in comparison to the control growth (*P* > 0.05) (Figure 5D). These results corroborate capsule analyses, since the capsule polysaccharides contribute considerably to the negative cell charge (Johnston and May, 2013).

### Thermodynamics Data

**FIGURE 6:**
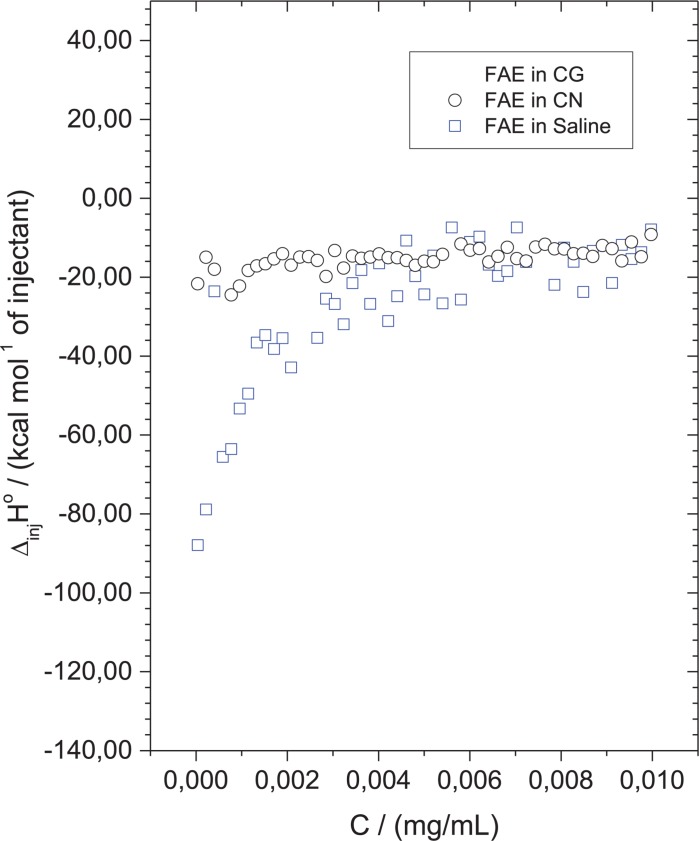
Ethyl acetate fraction (EAF) did not interact significantly with the surface of cryptococcal cells. Calorimetric titration curve for the dilution of concentrated EAF into saline solution (control) (blue square), *Cryptococcus gattii* (black squares), and *Cryptococcus neoformans*. Each titration experiment consisted of 51 successive injections of 5 μl of EAF at 1,000 mg/L in 1.5 ml of cell suspension at 1 × 10^6^ CFU/ml.

Aiming to analyze the molecular interactions between the EAF and cryptococcal cells, in this process, enthalpy changes were determined by ITC. Figure 6 shows that the interaction of the EAF with cryptococcal cells did not cause alterations in enthalpy, since the values of the test groups were similar to those of the blank experiment (EAF in saline).

### Cytotoxicity Assay

**FIGURE 7:**
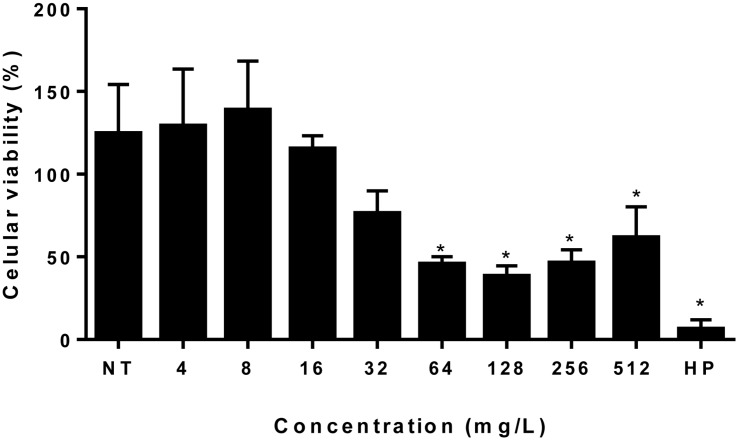
Ethyl acetate fraction cytotoxicity assay. Each bar represents the mean + standard deviation of the percentage of cell viability expressed in percentage of our independent experiments performed in triplicate considering the viability of untreated cells as 100%. *Statistical significance (*P* > 0.05). EAF, Ethyl acetate fraction; NT, No treatment; HP, Hydrogen peroxide.

MTT assay was performed in order to examine the cytotoxic effect of the EAF against PBMCs. These cells were treated with EAF in different concentrations, ranging from 4 to 512 mg/L. No significant cytotoxic effect was observed in normal PBMC at concentrations from 4 to 32 mg/ml (Figure 7).

## Discussion

Our results showed promising values of MIC for EAF against *C. gattii* and *C. neoformans*, since values ranged from 0.5 to 16 mg/l for both species. According to Ríos and Recio (2005), concentrations below 100 μg/ml for extracts and below 10 μg/ml for isolated compounds can be considered promising sources of substances with antimicrobial activity, opening new possibilities for the discovery of active molecules for the treatment of fungal infections. Newman and Cragg (2016), in a study evaluating the origin of drugs approved by the Food and Drug Administration (FDA) in the last 34 years, indicate that among the 221 new antimicrobials approved in that period, about 67% are related to natural products. Since currently available antifungal drugs do not fully meet the clinical needs either for the development of resistance or for high toxicity, it is necessary to continuously search for new chemical entities for the treatment of infections. In this context, natural products and derivatives are an invaluable source of substances with biological potential.

The MFC values of EAF against *C. gattii* and *C. neoformans* were similar to MIC values, ranging from 2 to 16 mg/L (Figure 2A), with an MFC/MIC ratio between 1 and 2. These results for antifungal tests or assays suggest that the EAF had a fungicidal effect on all tested yeasts strains (Peixoto et al., 2017). Time–kill curves demonstrated that the EAF kills cryptococcus cells in a time–concentration-dependent manner, similar to what can be seen in time–kill curves of AMB described by Burgess and Hastings (2000) and Cantón et al. (2004). It is important to note that no significant cytotoxic effect was observed in normal PBMCs in concentrations from 4 to 32 mg/ml that significantly affected cryptococcal cells (MIC ranged from 1 to 8 mg/L), suggesting that the effect of the EAF was more selective for yeasts than for PBMCs.

Another important point is that the analysis of the combination between FLC/AMB and EAF *in vitro* suggests that EAF does not impair the action of these drugs, but more studies are necessary to confirm this hypothesis.

To our knowledge, there are no studies about antimicrobial activity of *X. prockia* leaves, and there are just a few concerning other species of *Xylosma*, and these are limited to MIC evaluation. The antimicrobial activity of aqueous and alcoholic extracts of *Xylosma longifolium* leaves was observed against *Staphylococcus aureus*, *Bacillus subtilis*, and *Candida albicans* (Parveen and Ghalib, 2012). Other authors showed that *X. longifolium* methanol leaf extract has an inhibitory effect against *Trichophyton ajelloi* MTCC 4878 (140.62 mg/L) (Devi et al., 2013), with a much higher MIC than that observed for the EAF of *X. prockia* in our study (≤16 mg/ml).

Our results pointed that *X. prockia* EAF has hydroxycinnamic acid derivatives as majority compounds. Interestingly, although phenolic compounds are usually reported as free radical scavengers, our data demonstrate that EAF stimulates the production of intracellular oxidative and nitrosative species in cryptococcal cells. After 1 and 24 h of treatment with EAF, a significant oxidative burst was observed, but no modifications in mitochondrial membrane potential (Supplementary Figure S3) of yeast cells from both species. It is important to note that the straight connection between the ROS and the mitochondrial membrane potential is absent (Zhang et al., 2015). Several studies demonstrated that polyphenols can act as prooxidants under certain conditions, producing free radicals and causing cell injury (Eghbaliferiz and Iranshahi, 2016). The prooxidant activity of hydroxycinnamic acids was observed in the presence of Cu(II) with DNA damage (Zheng et al., 2008). It has been shown that curcumin- and resveratrol-mediated apoptosis is related to the increase in the concentrations of ROS (Heo et al., 2018). Epigallocatechin gallate from green tea can cause oxidative stress-related responses in *Saccharomyces cerevisiae* and can produce H_2_O_2_ in a weak alkaline medium (Maeta et al., 2007).

Therefore, we verified if EAF could induce lipid peroxidation in cryptococcal cells. TBARS levels were significantly high when both yeasts were treated with this fraction. The literature demonstrated considerable differences between these two species concerning the adaptation to stress condition (Jain and Fries, 2008; Hansakon et al., 2019). For example, Hamilton and Holdom (1997) demonstrated that Cu and Zn superoxide dismutase activity was present in supernatants of stationary-phase cultures of *C. neoformans* isolates, but undetectable in culture supernatants from *C. gattii* isolates.

We then assessed the ability of the EAF to modify the cryptococcal membrane. The lipid composition of the fungal cell membrane is predominantly composed of sterols, glycerophospholipids, and sphingolipids, and this structure is the most common target of anticryptococcal drugs (Sant et al., 2016). Interestingly, we observed that EAF impairs the cell membrane (Figure 5A), with a reduction of ergosterol content (Figure 4B). It is important to note that experiments with sorbitol indicate that this osmotic protectant does not impair the activity of the EAF against yeasts, since the MIC did not vary.

As far as we know, there is no study on the effect of phenolic acid derivatives on the ergosterol composition or biosynthesis, but data about mode of action of several other phenolic compounds provide some clues. Eugenol, methyl-eugenol, epigallocatechin-3-gallate, thymol, and carvacrol cause a reduction in ergosterol amounts in *Candida*, affecting the cell membrane (Navarro-Martínez et al., 2006; Ahmad et al., 2010, 2011).

To better understand how cryptococcal cells adapt to EAF stress, we performed some experiments exposing yeasts cells in subinhibitory concentrations of EAF (0.5 × MIC). EAF induced damage to the cell membrane and diminished its size with an increase in the S/V ratio. *Cryptococcus* cells are plastic, since they can modify their morphotypes (yeast, pseudohypha, or hypha) or sizes (micro or giant cells), depending on environmental factors (Navarro-Martínez et al., 2006; May et al., 2016). Small cells, and, consequently, the high S/V ratio, adapt quickly to changes in stress conditions. Researchers observed that small cells were adapted for growth in the presence of macrophages (Feldmesser et al., 2001), azoles (Nosanchuk et al., 1999; Ferreira et al., 2015), AMB (Nosanchuk et al., 1999), and terbinafine (Guerra et al., 2012). However, other studies have shown that capsule growth has a high energy cost for the cell (Ferreira et al., 2015), and this can explain why the cells do not expand the capsule in the presence of EAF. Due to the capsule’s contribution to the negative charge of *Cryptococcus* cells (Nosanchuk and Casadevall, 1997), we did not find alterations in the ZP.

The binding forces between molecules and ligands may include electrostatic interactions, hydrogen bonds, Van der Waals interactions, and hydrophobic interactions. Thermodynamic parameters of the binding reactions reveal a valuable insight into the types of forces involved (Du et al., 2016). Our thermodynamic ITC data showed very low differences of enthalpies between EAF/yeasts and EAF/blank titrations, demonstrating that molecular interactions between the EAF and the surface of cryptococcal cells are very weak. This suggests that cell death caused by EAF in *C. gattii* and *C. neoformans* might be mainly mediated by intracellular target(s), probably through oxidative burst. However, further studies are needed to confirm this hypothesis.

## Data Availability Statement

All datasets generated for this study are included in the article/Supplementary Material.

## Author Contributions

MS and AS executed, analyzed, and interpreted the data for plant extraction and fractionation. IR, GS, and GC executed, analyzed, and interpreted the data for liquid chromatography and mass spectrometry. MF, GF, JCS, JR, EF, GJF, and AM executed, analyzed, and interpreted the data for tests about morphologic cell surface, ergosterol content, cellular wall integrity, lipid peroxidation, and oxidative burst from the interaction of EAF with cryptococcal cells. LS, JRS, WN, and EF executed, analyzed, and interpreted the data for tests about lysosomal and mitochondrial integrity from the interaction of EAF with cryptococcal cells. ÂD executed, analyzed, and interpreted the data for tests about thermodynamic changes from the interaction of eugenol with cryptococcal cells. LM executed, analyzed, and interpreted the data for tests about cytotoxicity. KL, GF, and DS conceptualized or designed all the work, prepared the article and translated into English.

## Conflict of Interest

The authors declare that the research was conducted in the absence of any commercial or financial relationships that could be construed as a potential conflict of interest.

## Footnotes

1 http://rsb.info.nih.gov/ij/
