# Supplementary material for: Evaluation of in vitro Antifungal Activity of Xylosma prockia (Turcz.) Turcz. (Salicaceae) Leaves Against Cryptococcus spp
Source: Front Microbiol. 2020 Feb 6;10:3114. doi: 10.3389/fmicb.2019.03114 (PMC7015862; doi:10.3389/fmicb.2019.03114)
Supplement: Supplementary file 1 [file Data_Shee_1.docx]

Supplementary material

Table S1. Screening of antimicrobial effects of ethanolic extract and organic fractions of *X. prockia* leaves against *C. gattii* and *C. neoformans*.

| Strains | MIC EE  (mg/L) | MIC HF  (mg/L) | MIC DF  (mg/L) | MIC EAF  (mg/L) | MIC BF  (mg/L) | MIC FLC  (mg/L) | MIC AMB  (mg/L) |
| --- | --- | --- | --- | --- | --- | --- | --- |
| *C. gattii* |  |  |  |  |  |  |  |
| 23/10993 (C) | 8 | 2 | 1 | 0.5 | 1 | 16 | 0.5 |
| 547/OTTI/94-PI-10 (A) | 32 | 32 | 2 | 2 | 2 | 16 | 0.5 |
| *C. neoformans* |  |  |  |  |  |  |  |
| ATCC 24067 | 8 | 8 | 2 | 1 | 1 | 4 | 0.5 |
| 96806 (C) | 8 | 8 | 2 | 0.5 | 2 | 8 | 0.5 |

Ethanolic extract (EE), hexane fraction (HF), dichlorometane fraction (DF), ethyl acetate fraction (EAF), n-butanol fraction (BF), fluconazole (FLC) and amphotericin B (AMB).

Figure S1 - Proposed fragmentation for each type of compound in ESI (-)

A

C

B

D

NT

MIC

NT

MIC


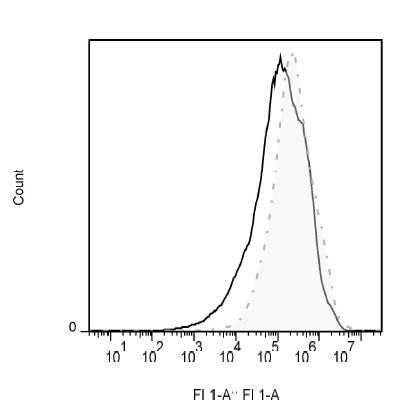


NT

MIC

NT

MIC

Figure S2 – EAF did not change mitochondrial membrane depolarization of the four strains *C. gattii* 32068 (A), *C. gattii* L27/01 (C), *C. neoformans* 28957 (B) and *C. neoformans* H99 (D) after 24 h of treatment. Data in graphs are represented in arbitrary units of fluorescence (AU) as the mean ± S.E. Data in histograms are represented in count in FL1. An asterisk represents statistical differences between the treatments and the control * (p <0.05). NT = No treatment; MIC = Minimal Inhibitory Concentration.
